# Multidisciplinary Delphi Consensus on the Clinical Use of Intranasal Carboxymethyl-β-Glucan-Resveratrol: Evidence-Based Recommendations for Upper Airway Diseases

**DOI:** 10.3390/jcm15083087

**Published:** 2026-04-17

**Authors:** Giorgio Ciprandi, Germano Bettoncelli, Ignazio La Mantia, Paola Mastromarino, Michele Miraglia del Giudice, Giovanni Arturo Rossi, Oliviero Rossi, Matteo Gelardi, Attilio Varricchio

**Affiliations:** 1 Department of Medicine and Health Sciences, University of Molise, 86100 Campobasso, Italy; attilio.varrichio@unimol.it; 2 Board of Physicians of Brescia, 25035 Brescia, Italy; germano.bettoncelli@gmail.com; 3 Faculty of Medicine and Surgery, University of Enna “Kore”, 94100 Enna, Italy; igolama@gmail.com; 4 Department of Public Health and Infectious Diseases, University of Rome “Sapienza”, 00185 Rome, Italy; paola.mastromarino@fondazione.uniroma1.it; 5 Department of Woman, Child and General and Specialized Surgery, University of Campania “Luigi Vanvitelli”, 80131 Naples, Italy; michele.miragliadelgiudice@unicampania.it; 6 Pediatric Pulmonology and Respiratory Endoscopy Unit, Istituto di Ricovero e Cura a Carattere Scientifico (IRCCS) Istituto Giannina Gaslini, 16147 Genova, Italy; giovannirossi@gaslini.org; 7 Immunoallergology Unit, University Hospital of Careggi, 50141 Florence, Italy; oliviero.rossi@unifi.it; 8 Faculty of Medicine, University of Foggia, 71122 Foggia, Italy; matteo.gelardi@unifg.it

**Keywords:** Delphi consensus, resveratrol, carboxymethyl-β-glucan, respiratory infections, allergic rhinitis, upper airway diseases

## Abstract

**Background/Objectives:** Intranasal carboxymethyl-β-glucan (CMBG)-resveratrol represents an innovative therapeutic approach for upper airway diseases, combining antimicrobial, anti-inflammatory, antioxidant, immunomodulatory, and antiallergic properties. Despite growing preclinical and clinical evidence, consensus on its clinical applications remains poorly defined. To establish evidence-based recommendations for the clinical use of intranasal CMBG-resveratrol through a multidisciplinary Delphi Consensus process. **Methods:** A two-round Delphi Consensus was conducted. In the first round, an expert board prepared, reviewed, and validated 22 statements based on current scientific evidence from preclinical and clinical studies. In the second round, 38 multidisciplinary experts evaluated each statement using a 5-point Likert scale (from 5 = strongly agree to 1 = strongly disagree). Consensus was defined as ≥80% agreement (scores 4 + 5). **Results:** All 22 statements achieved consensus (range: 83–100%). Strong agreement (≥90%) was reached for statements regarding the pathophysiological rationale (infection-inflammation-oxidative stress cycle), resveratrol’s pleiotropic mechanisms of action, the role of CMBG in enhancing stability and bioavailability, and clinical efficacy in respiratory infections and allergic rhinitis. The mean scores ranged from 4.2 to 4.9, indicating high expert agreement across all domains. **Conclusions:** This multidisciplinary Delphi Consensus provides evidence-based recommendations for the use of intranasal CMBG-resveratrol to manage upper airway diseases, particularly respiratory infections and allergic rhinitis. The formulation’s multitarget approach addresses the complex pathophysiology of these conditions through simultaneous antimicrobial (mainly antiviral), anti-inflammatory, and immunomodulatory effects.

## 1. Introduction

Upper airway diseases, including respiratory infections and allergic rhinitis, represent a significant global health burden affecting millions of individuals worldwide. These conditions share complex pathophysiological mechanisms involving immune system activation, inflammatory responses, and oxidative stress, creating self-perpetuating cycles that amplify disease severity and duration [1,2]. The management of these conditions requires therapeutic approaches that simultaneously address multiple pathogenic pathways.

Resveratrol (3,5,4′-trihydroxy-trans-stilbene) is a non-flavonoid polyphenol that functions as a phytoalexin, produced naturally by plants in response to pathogen attacks and environmental stress [3]. This defensive nature underlies its remarkable pleiotropic biological activities, including antimicrobial (mainly antiviral), anti-inflammatory, antioxidant, immunomodulatory, and antiallergic properties [4,5]. These multifaceted mechanisms make resveratrol a promising therapeutic candidate for upper airway diseases. However, oral resveratrol exhibits poor bioavailability due to rapid metabolism and limited absorption, necessitating high doses that may cause adverse effects [6,7]. To overcome these limitations, an innovative intranasal formulation combining trans-resveratrol with carboxymethyl-β-glucan (CMBG) has been developed [8]. The CMBG serves dual functions: it enhances resveratrol stability and solubility in aqueous solutions while exerting immunomodulatory effects as a biological response modifier [9,10]. This combination enables effective topical delivery to the nasal mucosa with controlled release, optimizing therapeutic efficacy while minimizing systemic exposure. At the molecular level, the combination of CMBG and resveratrol acts through complementary and synergistic mechanisms. Resveratrol exerts antiviral activity primarily through inhibition of viral replication and suppression of NLRP3 inflammasome activation; anti-inflammatory effects via NF-κB pathway inhibition and reduced pro-inflammatory cytokine production; antioxidant activity through reactive oxygen species (ROS) scavenging; immunomodulatory effects via SIRT1 deacetylase activation; and antiallergic activity through mast cell stabilization and reduction in type 2 inflammation markers. CMBG, beyond its role as a bioadhesive carrier that enhances resveratrol stability, aqueous solubility, and controlled mucosal release, exerts its own immunomodulatory activity as a biological response modifier, including induction of trained immunity through epigenetic reprogramming of innate immune cells. This mechanistic complementarity provides the scientific rationale for the combination formulation.

Preclinical studies have demonstrated that CMBG-resveratrol maintains long-term stability, inhibits rhinovirus replication, reduces pro-inflammatory cytokine expression (IL-6, IL-8, RANTES), and decreases ICAM-1 expression on epithelial cells [11,12]. ICAM-1 plays a critical role in both allergic inflammation, where it facilitates eosinophil infiltration, and viral infections, serving as the primary receptor for rhinoviruses [13,14]. Subsequently, several controlled clinical trials have evaluated intranasal CMBG-resveratrol in children and adults with respiratory infections, allergic rhinitis, and post-surgical recovery, demonstrating consistent efficacy and safety [15,16,17].

Despite this growing body of evidence, comprehensive consensus recommendations for the clinical use of intranasal CMBG-resveratrol remain poorly defined. The Delphi method represents a well-established approach for achieving expert consensus on clinical topics where evidence requires interpretation and integration into practice recommendations [18]. This structured process enables multidisciplinary experts to systematically evaluate scientific evidence, facilitating the development of evidence-based clinical guidance.

The present iterative initiative aimed to establish multidisciplinary consensus on the clinical use of intranasal CMBG-resveratrol through a rigorous two-round Delphi process. The first round involved an expert board that prepared, developed, and validated statements based on current scientific evidence. The second round engaged multidisciplinary experts who evaluated these statements to determine the level of agreement. This consensus provides clinicians with evidence-based recommendations for incorporating intranasal CMBG-resveratrol into the management of upper airway diseases.

## 2. Methods

### 2.1. Study Design

This study employed an iterative two-round Delphi consensus methodology to establish evidence-based recommendations for the clinical use of intranasal CMBG-resveratrol. The Delphi technique is a structured communication method that relies on a panel of experts to achieve convergence of opinion on specific issues through iterative rounds of questionnaires [19]. This approach is particularly valuable when empirical evidence requires expert interpretation and integration into clinical practice guidelines. So, the Delphi process is a systematic approach to gathering and consolidating expert opinions, ensuring a robust foundation for clinical recommendations.

### 2.2. First Round: Expert Board Statement Development

The first round involved an expert board composed of eight specialists in allergology, immunology, pediatrics, otorhinolaryngology, pulmonology, and microbiology. This multidisciplinary panel conducted a comprehensive review of the current scientific literature on resveratrol, CMBG, and their combination delivered intranasally. The literature review encompassed preclinical studies investigating molecular mechanisms, pharmacokinetic properties, and in vitro efficacy, as well as clinical trials evaluating therapeutic outcomes in various upper airway conditions.

Based on this evidence synthesis, the expert board developed 22 statements covering five key domains: pathophysiological rationale for upper airway diseases, resveratrol’s mechanisms of action, the role of CMBG in formulation optimization, preclinical evidence, and clinical efficacy (Table 1). Each statement was carefully formulated to reflect current scientific understanding while maintaining clinical relevance. The expert board achieved unanimous agreement on all 22 statements, confirming their scientific validity and clinical applicability. These five domains guided the expert board’s comprehensive assessment and the formulation of statements.

### 2.3. Second Round: Multidisciplinary Expert Evaluation

The second round engaged 38 experts from multiple medical specialties, selected based on their scientific background, clinical expertise and experience in managing upper airway diseases. The expert panel included allergologists, immunologists, pediatricians, otorhinolaryngologists, and general practitioners. This diverse composition ensured comprehensive evaluation from various clinical perspectives.

Each expert independently evaluated all 22 statements using a 5-point Likert scale: 5 = strongly agree, 4 = agree, 3 = neither agree nor disagree, 2 = disagree, 1 = strongly disagree. Experts were provided with supporting scientific references for each statement to facilitate informed evaluation. The questionnaire was administered electronically to ensure standardized data collection and maintain expert anonymity, using the web platform SurveyMonkey (https://it.surveymonkey.com).

### 2.4. Consensus Definition and Statistical Analysis

Consensus was defined a priori as ≥80% agreement, calculated as the proportion of experts rating a statement as 4 or 5 on the Likert scale. This threshold is consistent with established Delphi methodology standards [20]. For each statement, we calculated the percentage agreement (scores 4 + 5), the mean score, and the standard deviation (SD). Descriptive statistics were used to summarize expert responses across all statements and domains. In addition to percentage agreement, mean score, and standard deviation, a semi-quantitative analysis was performed for each statement, including calculation of the median score, interquartile range (IQR), and frequency distribution of each response category [1,2,3,4,5].

The consensus process did not require a third round, as all statements achieved the predefined consensus threshold in the second round. Results were analyzed to identify areas of strongest agreement and to characterize the overall level of expert consensus regarding the clinical use of intranasal CMBG-resveratrol.

To provide a more granular characterization of expert agreement, a semi-quantitative analysis was performed using the full Likert scale distribution (1 = strongly disagree to 5 = strongly agree) for each statement. For each statement, the frequency distribution of each response category, the median score, and the interquartile range (IQR) were calculated and are reported in the updated Table 1. Median scores were 5 (strongly agree) for 18 out of 22 statements, and 4 (agree) for the remaining 4 statements (Statements 12, 13, 17, 21, and 22), reflecting the strong positive skew of the distribution. IQR values were consistently narrow (4–5 for all statements, and 5–5 for Statements 2, 3, 7, and 8), confirming the strong convergence of expert opinion across all domains. Scores of 1 (strongly disagree) were recorded for only 3 statements (Statements 4, 12, 17, and 20), each by a single expert, indicating minimal dissent. These findings corroborate the high percentage agreement and mean scores reported above, and further support the robustness of the consensus achieved.

### 2.5. Ethical Considerations

This consensus study involved expert opinion evaluation and did not include patient data or interventions. The study was conducted in accordance with principles of scientific integrity and transparency. Regarding the clinical studies cited in this manuscript, all were conducted in Italy and received approval from the relevant Institutional Ethics Committees, as documented in each original publication. These studies were conducted within the regulatory framework applicable to CE-marked medical devices (EU MDR 2017/745) and were not performed under an Investigational New Drug (IND) application, as the CMBG-resveratrol formulation is marketed in Italy as a medical device.

## 3. Results

### 3.1. Overview of Consensus Achievement

**Figure 1:**
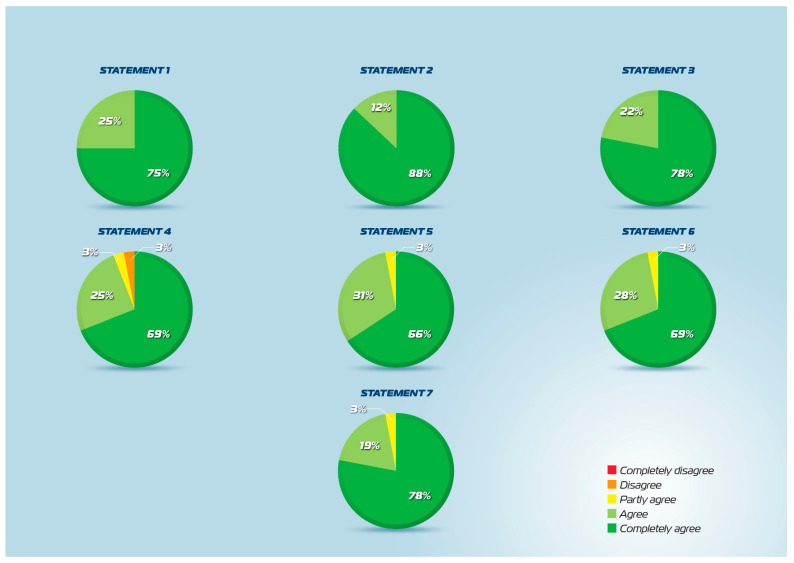
Statements 1–7 agreement scores.

**Figure 2:**
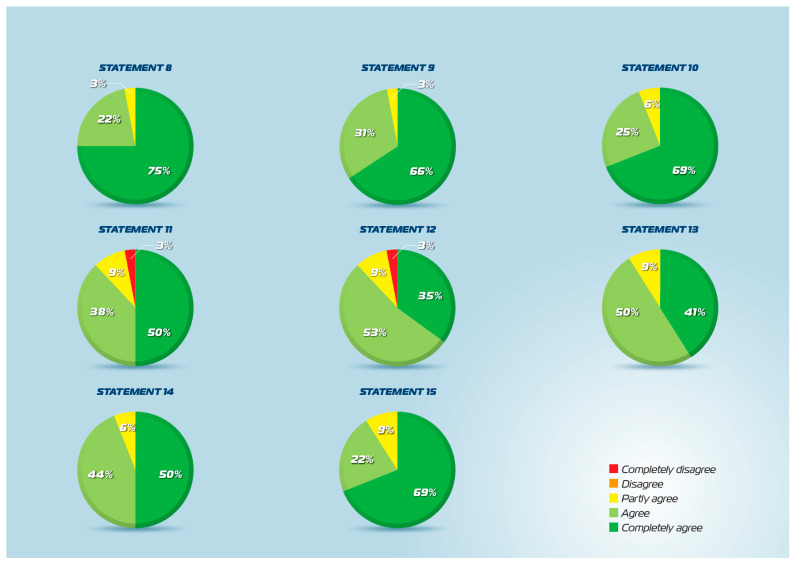
Statements 8–15 agreement scores.

**Figure 3:**
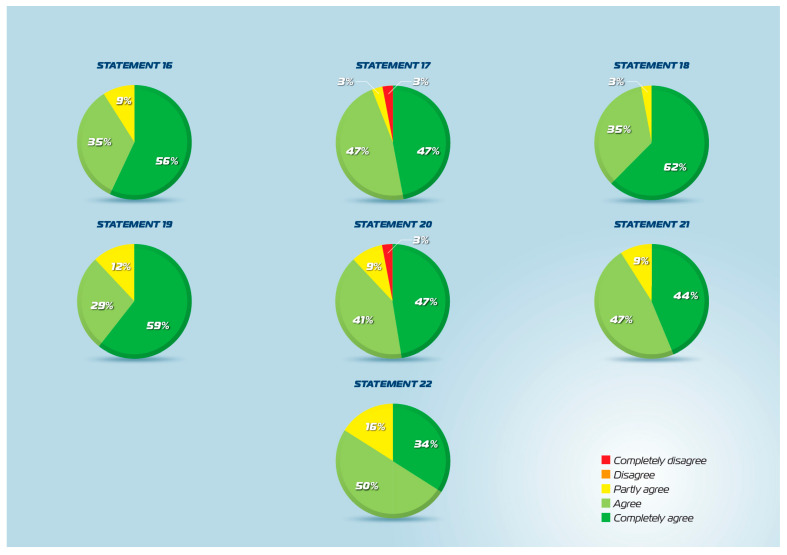
Statements 15–22 agreement scores.

All 22 statements achieved the predefined consensus threshold of ≥80% agreement in the second round of the Delphi process (Figure 1, Figure 2 and Figure 3). The percentage of agreement ranged from 83% to 100%, with mean scores ranging from 4.2 to 4.9 on the 5-point Likert scale. Standard deviations were consistently low (0.2–0.9), indicating strong convergence of expert opinion across all domains. These results demonstrate robust multidisciplinary consensus supporting the clinical use of intranasal CMBG-resveratrol for the treatment of upper airway diseases.

### 3.2. Pathophysiological Rationale (Statements 1–8)

The statements addressing the pathophysiological basis of upper airway diseases achieved exceptionally high consensus. Statement 1, describing how acute infectious processes activate the immune system and trigger inflammatory responses that can progress to hyperinflammation, achieved 100% agreement with a mean score of 4.8 (SD 0.4). Similarly, Statement 2, addressing the role of oxidative stress in amplifying inflammatory responses following infection, achieved 100% agreement with a mean score of 4.9 (SD 0.2), the highest across all statements. Statements 3 and 4 addressed allergic respiratory diseases and type 2 inflammation. Statement 3, describing type 2 immune polarization and eosinophil infiltration in allergic diseases, achieved 100% agreement (mean 4.8, SD 0.4). Statement 4, linking type 2 allergic inflammation to oxidative stress and identifying fractional exhaled nitric oxide (FeNO) as a biomarker, achieved 93% agreement (mean 4.7, SD 0.3). The relationship between allergy and infection susceptibility was addressed in Statements 5 and 6. Statement 5, indicating that allergic individuals experience more frequent, severe, and prolonged respiratory infections due to type 2 polarization, achieved 97% agreement (mean 4.6, SD 0.6). Statement 6, explaining ICAM-1 overexpression in allergic subjects and its dual role in inflammatory infiltration and increased infection susceptibility, achieved 97% agreement (mean 4.6, SD 0.5). Statements 7 and 8 synthesized the pathophysiological concepts. Statement 7, describing the vicious cycle involving immune activation, inflammation, and oxidative stress in both infections and allergies, achieved 97% agreement (mean 4.7, SD 0.5). Statement 8, noting that type 2 infection and inflammation amplify each other during the acute phase, also achieved 97% agreement (mean 4.7, SD 0.5).

### 3.3. Resveratrol: Mechanisms of Action (Statements 9–13)

Statements addressing resveratrol’s properties and mechanisms achieved a strong consensus. Statement 9, defining resveratrol as a phytoalexin produced by plants for defense against microbial stimuli, achieved 97% agreement (mean 4.6, SD 0.6). Statement 10, describing resveratrol’s pleiotropic activities, including antimicrobial, antioxidant, immunomodulatory, anti-inflammatory, and antiallergic effects, achieved 93% agreement (mean 4.6, SD 0.6). Statement 11 concerning the potential use in clinical practice achieved 94% agreement (mean 4.3, SD 0.9). Statement 12, addressing the limitation of poor oral bioavailability requiring high doses that may cause side effects, achieved 87% agreement (mean 4.2, SD 0.9). Statement 13, describing the development of formulations ensuring adequate stability and solubility for clinical efficacy at low doses, achieved 93% agreement (mean 4.3, SD 0.6).

### 3.4. CMBG-Resveratrol Formulation (Statements 14–16)

Statements addressing the CMBG-resveratrol formulation achieved a strong consensus. Statement 14, describing how CMBG addition provides chemical-physical characteristics enabling mucosal delivery with controlled release suitable for nasal administration, achieved 93% agreement (mean 4.4, SD 0.6). Statement 15, proposing intranasal CMBG-resveratrol as a suitable option for managing respiratory infections, especially viral and allergic diseases, achieved 90% agreement (mean 4.6, SD 0.7). Statement 16, describing CMBG’s dual functions of stabilizing resveratrol for topical availability and stimulating the immune system, including trained immunity, achieved 90% agreement (mean 4.5, SD 0.7). These results confirm expert recognition of CMBG’s critical role in optimizing resveratrol delivery and enhancing therapeutic efficacy through its own immunomodulatory properties.

### 3.5. Preclinical Evidence (Statements 17–18)

The preclinical evidence statements achieved high consensus. Statement 17, confirming that in vitro studies demonstrated formulation stability, water solubility, and suitability for nebulization, achieved 93% agreement (mean 4.3, SD 0.8). Statement 18, describing preclinical confirmation of antiviral and anti-inflammatory activity through reduced ICAM-1 expression and pro-inflammatory cytokines, achieved 97% agreement (mean 4.6, SD 0.5). These results indicate strong expert acceptance of the preclinical foundation supporting clinical translation of intranasal CMBG-resveratrol. The strong consensus on Statement 18 particularly underscores the recognition of mechanistic evidence linking formulation properties to therapeutic effects.

### 3.6. Clinical Evidence (Statements 19–22)

The clinical efficacy statements achieved consensus ranging from 83% to 90%. Statement 19, confirming that controlled clinical studies demonstrated CMBG-resveratrol’s ability to improve the clinical course of respiratory infections, especially viral, achieved 87% agreement (mean 4.5, SD 0.7). Statement 20, indicating that clinical studies demonstrated infection-prevention capability, particularly for viral infections, in individuals with recurrent respiratory infections, achieved 87% agreement (mean 4.3, SD 0.9). Statement 21, describing clinical demonstration of symptom reduction in allergic rhinitis patients with concurrent reduction in type 2 inflammation, achieved 90% agreement (mean 4.3, SD 0.7). Statement 22, proposing utility in post-operative recovery following endonasal surgery, achieved 83% agreement (mean 4.2, SD 0.7), representing the lowest consensus level among all statements, though still exceeding the predefined threshold. The clinical efficacy statements, while achieving consensus, showed slightly lower agreement rates and higher standard deviations than pathophysiological and mechanistic statements. This pattern likely reflects a more limited clinical evidence base than preclinical data, as well as the inherent variability in clinical practice across the diverse expert panel.

No statement failed to achieve consensus, and no third Delphi round was required. The consistently low standard deviations across all statements indicate strong convergence of expert opinion, supporting the robustness of these consensus recommendations.

## 4. Discussion

This multidisciplinary Delphi Consensus achieved robust agreement across all 22 statements addressing the clinical use of intranasal CMBG-resveratrol for upper airway diseases. It is important to contextualize these findings within the appropriate regulatory and evidentiary framework. The intranasal CMBG-resveratrol formulation is currently marketed in Italy as a CE-marked medical device under EU MDR 2017/745, and has been commercially available for several years. However, it should be explicitly noted that resveratrol is not approved as a pharmaceutical agent for intranasal use, and CMBG is not approved as a pharmaceutical excipient for intranasal administration. The safety and efficacy of this combination for upper airway application have not been established through Phase III randomized controlled trials meeting regulatory drug approval standards. The present Delphi consensus, while grounded in published preclinical and clinical evidence, represents expert opinion and should be interpreted as exploratory and hypothesis-generating rather than as established clinical guidelines. The findings are intended to provide clinicians with a structured synthesis of current evidence and to identify priorities for future research, including adequately designed, controlled clinical trials. The unanimous achievement of consensus thresholds, with agreement ranging from 83% to 100% and mean scores from 4.2 to 4.9, demonstrates strong expert support for this therapeutic approach. The consensus encompasses the pathophysiological rationale, molecular mechanisms, formulation optimization, preclinical evidence, and clinical efficacy, providing comprehensive evidence-based recommendations for clinical practice. A recent review on the subject served as the reference for this initiative [21].

### 4.1. Pathophysiological Rationale and Multitarget Approach

The exceptionally high consensus on pathophysiological statements (93–100% agreement) confirms expert recognition of the complex interplay between infection, inflammation, and oxidative stress in upper airway diseases. This understanding is critical because it establishes the scientific foundation for multitarget therapeutic approaches. The vicious cycle involving immune activation, inflammatory responses, and oxidative stress amplification creates self-perpetuating disease processes that single-target therapies may inadequately address [22,23]. The strong consensus on these concepts validates the rationale for interventions like CMBG-resveratrol that simultaneously target multiple pathogenic mechanisms.

### 4.2. Molecular Mechanisms of Resveratrol and CMBG

The high consensus on resveratrol’s pleiotropic mechanisms (93–97% agreement) reflects expert appreciation of its multitarget therapeutic potential. In particular, resveratrol exerts: (i) antimicrobial activity, particularly against viruses, addresses the infectious trigger of upper airway inflammation [24]; (ii) anti-inflammatory effects, mediated through NF-κB pathway inhibition and reduced pro-inflammatory cytokine production, dampen the inflammatory cascade [25,26]; (iii) antioxidant properties, neutralizing reactive oxygen and nitrogen species, breaking the oxidative stress-inflammation cycle [27,28]; (iv) immunomodulatory effects, including SIRT1 activation and trained immunity enhancement, optimize host defense responses [29,30]; and (v) antiallergic activity through mast cell stabilization and reduced type 2 inflammation addresses allergic components of upper airway disease [31,32]. This multitarget profile distinguishes resveratrol from conventional single-mechanism therapies. While antibiotics address bacterial infections and corticosteroids suppress inflammation, neither adequately addresses the full spectrum of pathogenic mechanisms in upper airway diseases. Resveratrol’s ability to simultaneously target infection, inflammation, oxidative stress, immune dysfunction, and allergic responses provides a more comprehensive therapeutic approach aligned with disease pathophysiology.

### 4.3. Formulation Optimization: The Role of CMBG

The strong consensus on CMBG’s role (90–93% agreement) highlights expert recognition that formulation optimization is essential for translating resveratrol’s biological activities into clinical efficacy. The poor oral bioavailability of resveratrol, resulting from rapid metabolism and limited absorption, has historically limited its therapeutic application [33,34]. High oral doses required to achieve therapeutic tissue concentrations increase the risk of adverse effects and reduce patient compliance. The CMBG-resveratrol formulation addresses these limitations through multiple mechanisms, including an enhanced resveratrol stability in aqueous solutions, preventing degradation and maintaining biological activity [8]; increased water solubility by the carboxymethylation of β-glucan, so enabling effective topical delivery [35]; prolonged mucosal contact, as CMBG provides bioadhesive properties that prolong mucosal contact time, facilitating controlled release and sustained therapeutic effects [36]; immunomodulatory activity, as CMBG itself exhibits immunomodulatory activities as a biological response modifier, contributing additional therapeutic benefits beyond its carrier function [10,37].

The concept of trained immunity, referenced in Statement 16, represents an emerging immunological paradigm with significant therapeutic implications. Trained immunity describes the enhanced responsiveness of innate immune cells following initial stimulation, mediated through epigenetic reprogramming [38]. β-glucans are potent inducers of trained immunity, enhancing macrophage and natural killer cell responses to subsequent challenges [39]. This mechanism may explain the preventive effects of CMBG-resveratrol observed in clinical studies of recurrent respiratory infections, where treatment reduced infection frequency and severity [16,40].

The intranasal route of administration offers additional advantages for the treatment of upper airway diseases. Topical delivery achieves high local concentrations at the site of pathology while minimizing systemic exposure and potential adverse effects [41]. The nasal mucosa provides rapid absorption and direct access to the respiratory tract, enabling prompt therapeutic action. Furthermore, intranasal administration bypasses first-pass hepatic metabolism, improving bioavailability compared to oral routes [42].

### 4.4. Preclinical and Clinical Evidence

The high consensus on preclinical evidence (93–97% agreement) and clinical efficacy (83–90% agreement) demonstrates expert confidence in the translational pathway from laboratory research to clinical application. Preclinical studies established that CMBG-resveratrol inhibits rhinovirus replication, reduces pro-inflammatory cytokine expression (IL-6, IL-8, RANTES), and decreases ICAM-1 expression on epithelial cells [11,12]. These mechanistic findings directly correlate with clinical outcomes observed in controlled trials. The reduction in ICAM-1 expression merits particular attention due to its dual significance in upper airway diseases. ICAM-1 serves as the primary receptor for major group rhinoviruses, which cause the majority of common colds [43]. Decreased ICAM-1 expression reduces viral binding and entry, explaining the antiviral clinical effects. Simultaneously, ICAM-1 functions as an adhesion molecule facilitating leukocyte recruitment to inflamed tissues [44]. In allergic rhinitis and conjunctivitis, ICAM-1 upregulation promotes eosinophil infiltration, amplifying type 2 inflammation [45]. By reducing ICAM-1 expression, CMBG-resveratrol simultaneously addresses both infectious and allergic components of upper airway inflammation.

Clinical trials have demonstrated consistent efficacy across diverse patient populations and clinical scenarios. In children with acute rhinopharyngitis and recurrent respiratory infections, CMBG-resveratrol significantly reduced symptom severity and rescue medication use compared to saline control [15]. In children with persistent allergic rhinitis and frequent infections, treatment reduced nasal and bronchial symptoms, fever duration, cough frequency, bronchodilator use, and school absence [16]. In infants with acute respiratory infections, CMBG-resveratrol reduced nasal symptom severity [17]. In children with recurrent respiratory infections, treatment reduced wheezing episodes, hospitalizations, and the need for oral corticosteroids [40]. In children with seasonal allergic rhinitis, CMBG-resveratrol treatment improved symptoms and decreased on-demand antihistamine use [46]. In adults with persistent allergic rhinitis, intranasal resveratrol improved symptoms and quality of life while significantly reducing IgE, IL-4, TNF-α, and eosinophil levels, with efficacy comparable to intranasal budesonide [47]. These findings confirm that CMBG-resveratrol addresses both symptomatic and inflammatory components of allergic disease.

The slightly lower consensus on clinical efficacy statements (83–90%) compared to pathophysiological and mechanistic statements (93–100%) likely reflects several factors. First, the clinical evidence base, while growing, remains more limited than preclinical data, with relatively few published randomized controlled trials. Second, clinical outcomes inherently exhibit greater variability than laboratory measurements due to patient heterogeneity, differences in disease severity, and diverse treatment protocols. Third, expert clinical experience varies across patient populations and practice settings. Nevertheless, all clinical efficacy statements achieved consensus, supporting the clinical utility of CMBG-resveratrol across multiple indications.

### 4.5. Safety Profile and Tolerability

The safety and local tolerability of intranasal CMBG-resveratrol have been evaluated across all published clinical trials, encompassing both pediatric and adult populations. In all studies, the formulation was well tolerated, with no serious adverse events reported. Local tolerability in the upper airways was specifically assessed in each trial, and no clinically significant local irritation, mucosal toxicity, or other adverse local effects were observed. Follow-up durations ranged from short-term acute treatment periods (approximately 10 days for acute respiratory infections) to longer preventive treatment periods (3–6 months for recurrent infections). Safety data were collected across diverse age groups, including infants, preschool children, school-age children, and adults, with no age-specific safety concerns identified. A comprehensive review of the safety profile of intranasal CMBG-resveratrol across all published studies has recently been published [21], to which the reader is referred for detailed safety data. These findings support the favorable tolerability profile of the formulation; however, long-term safety data and systematic pharmacovigilance studies remain limited and represent an important priority for future research.

### 4.6. Regulatory Status and Evidentiary Framework

The regulatory status of the CMBG-resveratrol formulation warrants explicit discussion. In Italy and the European Union, the product is classified and marketed as a medical device under EU MDR 2017/745, which requires demonstration of safety and performance prior to CE marking. This classification is distinct from pharmaceutical drug approval, which requires demonstration of efficacy and safety through controlled clinical trials under regulatory oversight (e.g., IND application in the USA, CTA in the EU). All clinical studies cited in this manuscript were conducted within the medical device regulatory framework and received approval from the relevant Institutional Ethics Committees in Italy, as documented in each original publication. It should be noted that the available clinical evidence, while consistent and promising, derives primarily from relatively small controlled trials, predominantly in pediatric populations. The evidence base does not yet meet the standards required for pharmaceutical drug approval. Therefore, the recommendations emerging from this Delphi consensus should be interpreted as expert-informed, evidence-based guidance to support clinical decision-making, while explicitly acknowledging the need for larger, adequately powered, randomized controlled trials to definitively establish the safety and efficacy of intranasal CMBG-resveratrol for upper airway diseases.

### 4.7. Clinical Implications and Practical Recommendations

This consensus provides evidence-based guidance for incorporating intranasal CMBG-resveratrol into clinical practice for upper airway diseases, as schematically represented in Figure 1.

For respiratory infections, particularly viral infections, CMBG-resveratrol may represent a rational therapeutic option that simultaneously targets multiple pathogenic mechanisms. The formulation may be particularly valuable for patients with recurrent respiratory infections, where its preventive effects through trained immunity induction offer advantages beyond acute symptom management.

For allergic rhinitis, CMBG-resveratrol may provide a multi-mechanistic approach that targets both allergic inflammation and the increased infection susceptibility characteristic of allergic patients. The reduction in type 2 inflammation markers, including IgE, IL-4, and eosinophils, demonstrates disease-modifying effects beyond symptomatic relief. The comparable efficacy to intranasal corticosteroids observed in one study, combined with the favorable safety profile, suggests CMBG-resveratrol may serve as an alternative or adjunctive therapy, particularly for patients seeking natural treatment options or experiencing corticosteroid-related adverse effects [47].

The consensus on post-surgical applications, while achieving the lowest agreement level (83%), indicates expert recognition of potential utility in this setting [48]. Post-operative inflammation and infection risk represent significant concerns following endonasal surgery. CMBG-resveratrol’s combined anti-inflammatory and antimicrobial properties, along with its potential wound-healing effects mediated by β-glucan, provide a rational basis for post-surgical use. However, the lower consensus level suggests that additional clinical evidence would strengthen recommendations in this indication.

This Delphi Consensus exhibits several methodological strengths. The two-round Delphi process followed established methodology with predefined consensus thresholds. The multidisciplinary expert panel of specialists ensured diverse clinical perspectives and comprehensive evaluation. The systematic development of statements based on current scientific evidence provided a solid foundation for expert evaluation. The achievement of consensus across all statements without requiring a third round demonstrates strong convergence of expert opinion.

However, several limitations warrant consideration. First, the Delphi method relies on expert opinion rather than direct empirical evidence, although all statements were grounded in published scientific data. Second, the clinical evidence base for CMBG-resveratrol, while growing, remains more limited than preclinical data, as reflected in the slightly lower consensus on clinical efficacy statements. Third, most clinical trials have been conducted in pediatric populations, with fewer studies in adults. Fourth, long-term efficacy and safety data remain limited, although available studies report favorable safety profiles. Fifth, the optimal dosing regimens, treatment duration, and patient selection criteria require further investigation through additional clinical trials.

On the other hand, this Delphi Consensus contributes to the growing recognition that complex diseases characterized by multiple pathogenic mechanisms may benefit from multitarget therapeutic approaches. The traditional pharmaceutical paradigm emphasizing single-target specificity, while valuable for many conditions, may prove insufficient for diseases involving intricate pathophysiological networks. Upper airway diseases exemplify this complexity, with infection, inflammation, oxidative stress, immune dysfunction, and allergic responses interacting in self-amplifying cycles. Therapeutic strategies addressing multiple mechanisms simultaneously, as demonstrated by CMBG-resveratrol, align more closely with disease pathophysiology and may achieve superior clinical outcomes compared to single-target interventions. The integration of natural compounds with established biological activities into evidence-based clinical practice represents an important trend in modern medicine. Resveratrol, derived from plants and consumed in various foods, exhibits a favorable safety profile supported by extensive preclinical and clinical data. The development of optimized formulations, such as CMBG-resveratrol, that overcome bioavailability limitations while preserving biological activity demonstrates how pharmaceutical science can enhance the therapeutic potential of natural compounds. This approach bridges traditional natural medicine and modern evidence-based practice, offering patients effective treatment options grounded in rigorous scientific evaluation.

## 5. Conclusions

**Figure 4:**
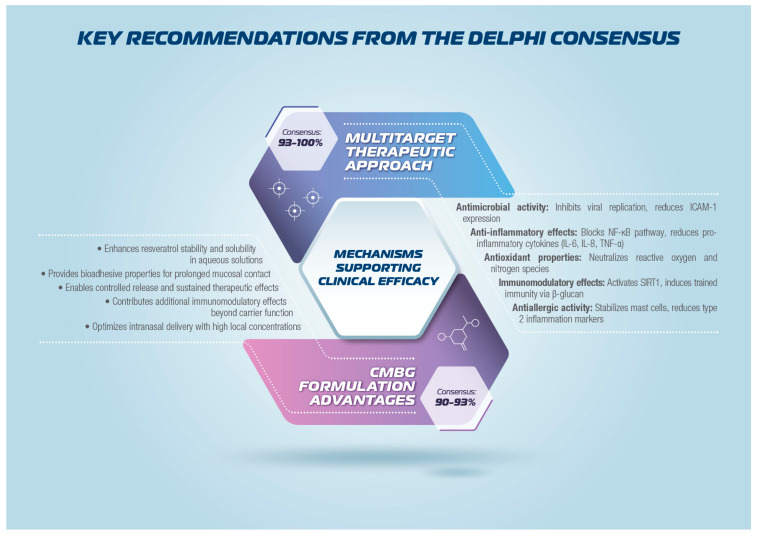
Key recommendations from the Delphi Consensus.

**Figure 5:**
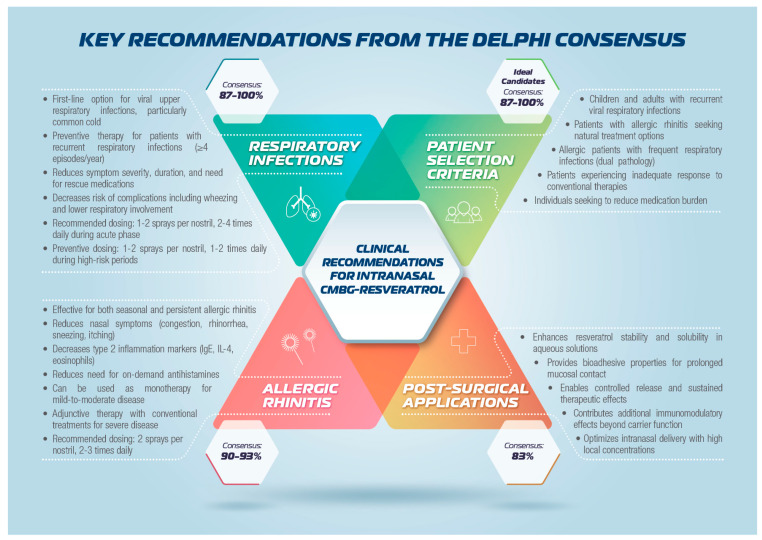
Key recommendations from the Delphi Consensus.

This multidisciplinary Delphi Consensus achieved robust agreement across all 22 statements addressing the clinical use of intranasal CMBG-resveratrol for upper airway diseases, with consensus ranging from 83% to 100% and mean scores from 4.2 to 4.9. The unanimous achievement of consensus thresholds demonstrates strong expert support for this therapeutic approach across pathophysiological rationale, molecular mechanisms, formulation optimization, preclinical evidence, and clinical efficacy domains. Figure 4 and Figure 5 summarize the key recommendations from the Delphi Consensus. The CMBG formulation plays a critical role in translating resveratrol’s biological activities into clinical efficacy by enhancing stability, solubility, and bioavailability while providing additional immunomodulatory effects through trained immunity induction. The intranasal route of administration optimizes local therapeutic concentrations while minimizing systemic exposure, offering advantages for the management of upper airway disease. In addition, resveratrol displays a relevant antiviral activity (mainly against influenza virus, respiratory syncytial virus, rhinovirus, and MERS-CoV) through different mechanisms, including inhibition of replication, NLRP3 inflammasome activation, promotion of autophagy processes, and activation of Sirtuin 1 deacetylase [21,49].

Therefore, this multidisciplinary Delphi consensus provides expert-supported, evidence-informed guidance on the potential clinical use of intranasal CMBG-resveratrol in upper airway diseases. It is important to emphasize that these findings are exploratory and hypothesis-generating in nature: resveratrol is not approved as a pharmaceutical agent for intranasal use, and the available clinical evidence, while promising, has not yet been established through large-scale, adequately powered randomized controlled trials. The formulation is currently marketed in Italy as a CE-marked medical device. The present consensus identifies priorities for future research, including the need for well-designed, controlled clinical trials to definitively characterize the safety, efficacy, optimal dosing, and patient selection criteria for this multitarget therapeutic approach.

## Figures and Tables

**Table 1:** Domains and statements included in the multidisciplinary Delphi Consensus on the use of CMBG-Resveratrol in clinical practice and relevant answers.

Statement	% Agreement (Scores 4 + 5)	Mean (SD)	Median (IQR)
*Pathophysiological Rationale*			
(1) Every acute infectious process activates the immune system and triggers an inflammatory response. These phenomena are aimed at eliminating the pathogen and restoring homeostasis. However, in conditions of defective immune function, the inflammatory reaction can progress, worsening the infection, and, if uncontrolled, can even evolve into a condition of hyperinflammation, sustained by a cytokine storm.	100%	4.8 (0.4)	5 (4–5)
(2) The inflammatory reaction that follows an infection also generates oxidative stress, which in turn, if left unchecked, amplifies and maintains the inflammatory state.	100%	4.9 (0.2)	5 (5–5)
(3) Respiratory allergic diseases are characterized by activation of the immune system oriented toward polarization 2. This immunological balance promotes type 2 inflammation, characterized by eosinophil infiltration.	100%	4.8 (0.4)	5 (5–5)
(4) Type 2 allergic inflammation also generates oxidative stress, which in turn amplifies allergic inflammation. A biomarker of oxidative stress in type 2 inflammation is alveolar FeNO.	93%	4.7 (0.3)	5 (4–5)
(5) Allergic individuals, having type 2 polarization, are more susceptible to respiratory infections, which are also more severe and longer lasting than in non-allergic individuals.	97%	4.6 (0.6)	5 (4–5)
(6) Allergic subjects have an overexpression of ICAM-1 adhesion molecules, which, on the one hand, explain the inflammatory infiltrative phenomena and, on the other hand, justify the increased susceptibility to infections, as ICAM-1 is the main receptor for most rhinoviruses.	97%	4.6 (0.5)	5 (4–5)
(7) Both respiratory infections and respiratory allergies are characterized by a vicious cycle involving activation of the immune system, an inflammatory response, and oxidative stress.	97%	4.7 (0.5)	5 (5–5)
(8) Type 2 infection and inflammation are conditions that, in their acute phase, tend to amplify each other.	97%	4.7 (0.5)	5 (5–5)
*Mechanisms of Action*			
(9) Resveratrol is a polyphenol that, being a phytoalexin, is produced by plants in response to various stimuli, especially of microbial origin, to defend the plant itself.	97%	4.6 (0.6)	5 (4–5)
(10) Resveratrol exerts its defensive capacity through various mechanisms of action, including antimicrobial (especially antiviral) activity, antioxidant activity, immunomodulatory activity, anti-inflammatory activity, and anti-allergic activity.	93%	4.6 (0.6)	5 (4–5)
(11) Thanks to this pleiotropic activity, resveratrol can be used advantageously in clinical practice for various diseases.	94%	4.3 (0.9)	4.5 (4–5)
(12) When taken orally, resveratrol has poor systemic bioavailability, so very high doses must be used, which can easily cause side effects.	87%	4.2 (0.9)	4 (4–5)
(13) Formulations have been designed and developed that provide adequate stability and solubility in order to ensure clinically relevant efficacy even at low doses.	93%	4.3 (0.6)	4.5 (4–5)
*CMBG-Resveratrol Formulation*			
(14) The addition of the bioadhesive mucopolymer carboxymethyl-β-glucan (CMBG) to resveratrol results in a series of chemical-physical characteristics that allow it to be delivered to the mucous membranes, thus enabling controlled release. This makes the compound suitable for nasal administration.	93%	4.4 (0.6)	4.5 (4–5)
(15) The intranasal route of the CMBG-resveratrol compound may be a suitable option for managing respiratory infections, especially those of viral etiology, and respiratory allergic diseases.	90%	4.6 (0.7)	5 (4–5)
(16) Carboxymethyl-β-glucan has two functions: one is to stabilize and make resveratrol available topically, and the other is to stimulate the immune system (typical of glucans), also activating “Trained Immunity.”	90%	4.5 (0.7)	5 (4–5)
*Preclinical Evidence*			
(17) A series of preclinical in vitro studies have demonstrated that this formulation is stable, water-soluble, and suitable for nebulization.	93%	4.3 (0.8)	4.5 (4–5)
(18) These preclinical studies confirmed antiviral and anti-inflammatory activity, including through reduced expression of ICAM-1 and pro-inflammatory cytokines.	97%	4.6 (0.5)	5 (4–5)
*Clinical Evidence*			
(19) A series of controlled clinical studies has demonstrated the ability of the CMBG-resveratrol combination to improve the clinical course of respiratory infections, especially those of a viral nature.	87%	4.5 (0.7)	5 (4–5)
(20) These clinical studies have shown that the combination of CMBG and resveratrol can also prevent infections, especially viral infections, in individuals with recurrent respiratory infections.	87%	4.3 (0.9)	4 (4–5)
(21) A clinical study has demonstrated the ability of the CMBG-resveratrol combination to reduce symptoms in patients with allergic rhinitis, while also reducing type 2 inflammation.	90%	4.3 (0.7)	4 (4–5)
(22) The CMBG-resveratrol complex may also be useful in the post-operative course of endonasal surgery.	83%	4.2 (0.7)	4 (4–5)

## Data Availability

The original contributions presented in this study are included in the article. Further inquiries can be directed to the corresponding author.
